# No requirement of perioperative glucocorticoid replacement in patients with endogenous Cushing’s syndrome – a pilot study

**DOI:** 10.1007/s12020-024-03832-1

**Published:** 2024-04-22

**Authors:** Christian Trummer, Marlene Pandis, Verena Theiler-Schwetz, Lisa Schmitt, Barbara Obermayer-Pietsch, Verena Gellner, Andrea Simon, Stefan Pilz

**Affiliations:** 1 Division of Endocrinology and Diabetology, Department of Internal Medicine, Medical University of Graz, Graz, Austria; 2 Endocrinology Lab Platform, Department of Internal Medicine and Department of Gynecology and Obstetrics, Medical University of Graz, Graz, Austria; 3 Department of Neurosurgery, Medical University of Graz, Graz, Austria; 4 Division of General, Visceral, and Transplantation Surgery, Department of Surgery, Medical University of Graz, Graz, Austria

**Keywords:** Perioperative glucocorticoid replacement, Cushing’s syndrome, Cushing’s disease, Hypercortisolism, Pituitary, Adrenal

## Abstract

**Purpose:**

Surgical therapy represents the first-line treatment for endogenous Cushing’s syndrome (CS). While postoperative glucocorticoid replacement is mandatory after surgical remission, the role of perioperative glucocorticoid therapy is unclear.

**Methods:**

We recruited patients with central or adrenal CS in whom curative surgery was planned and patients who underwent pituitary surgery for other reasons than CS as a control group. Patients did not receive any perioperative glucocorticoids until the morning of the first postoperative day. We performed blood samplings in the morning of surgery, immediately after surgery, in the evening of the day of surgery, and in the morning of the first and third postoperative day before any morning glucocorticoid intake. We continued clinical and biochemical monitoring during the following outpatient care.

**Results:**

We recruited 12 patients with CS (seven with central CS, five with adrenal CS) and six patients without CS. In patients with CS, serum cortisol concentrations <5.0 µg/dL (<138 nmol/L) were detected in the morning of the first and third postoperative day in four (33%) and six (50%) patients, respectively. Morning serum cortisol concentrations on the third postoperative day were significantly lower when compared to preoperative measurements (8.5 ± 7.6 µg/dL vs. 19.9 ± 8.9 µg/dL [235 ± 210 nmol/L vs. 549 ± 246 nmol/L], *p* = 0.023). No patient developed clinical or biochemical signs associated with hypocortisolism. During follow-up, we first observed serum cortisol concentrations >5.0 µg/dL (>138 nmol/L) after 129 ± 97 days and glucocorticoids were discontinued after 402 ± 243 days. Patients without CS did not require glucocorticoid replacement at any time.

**Conclusion:**

Perioperative glucocorticoid replacement may be unnecessary in patients with central or adrenal CS undergoing curative surgery as first-line treatment.

## Introduction

Endogenous Cushing’s syndrome (CS) is a rare endocrine disorder with an estimated annual incidence between 1.8 and 3.2 cases per million individuals [1, 2]. It is characterized by an excessive cortisol synthesis caused by either exaggerated production of adrenocorticotropic hormone (ACTH) from pituitary or ectopic tumors, or by autonomous adrenal overproduction of cortisol [3]. The most common cause of endogenous CS is ACTH-secreting pituitary lesions (also known as Cushing’s disease, CD), which comprise ~70% of all cases [1, 3]. Glucocorticoid excess is responsible for numerous clinical complications including obesity, impaired glucose metabolism, impaired immune function, dyslipidemia, musculoskeletal disorders, neuropsychiatric disorders, arterial hypertension, and increased risk for thromboembolism [4]. Overall, CS is associated with an increased mortality, mainly caused by cardiovascular and infectious diseases, that correlates with the duration of disease activity [4, 5]. Even though rapid treatment induction is of utmost priority in CS, time to diagnosis is often significantly delayed [6–8]. For both central (pituitary) and adrenal CS, surgical intervention (i.e., endoscopic transsphenoidal selective tumor resection or adrenalectomy, respectively) represents the optimal initial treatment modality [3, 9].

After successful pituitary or adrenal surgery, patients require mandatory glucocorticoid replacement until the hypothalamic–pituitary–adrenal (HPA) axis recovers, which may often take more than a year depending on CS subtype and surgical method [9, 10]. While postoperative glucocorticoid therapy is common practice, the need for perioperative glucocorticoid replacement remains a topic of debate and is handled with broad heterogeneity [11]. Some experts advocate for perioperative glucocorticoids in doses that exceed the usual replacement therapy to avoid symptoms of acute steroid withdrawal [12, 13], whereas others delay glucocorticoids for up to several days postoperatively under close observation [14, 15]. An approach favoring delayed glucocorticoid administration is supported by the half-life of circulating serum cortisol of 70–120 min [16] and an even longer cortisol tissue half-life of 8–12 h [17]. Thus, even if secretion of ACTH and/or cortisol would cease entirely after pituitary or adrenal surgery, one would not expect serum cortisol concentrations to fall to critically low levels within the first postoperative hours [18]. If perioperative glucocorticoid replacement could be omitted, this would facilitate perioperative clinical care of patients including screening for postoperative hypocortisolism, which is considered a positive predictor for surgical remission [18, 19].

Although some previous studies suggest that perioperative glucocorticoid replacement may be safely delayed in patients undergoing surgery in pituitary or adrenal CS [18, 19], there still exists considerable heterogeneity regarding perioperative glucocorticoid therapy due to limited evidence from postoperative studies addressing this issue [11]. To evaluate the necessity of perioperative glucocorticoid replacement, we designed this prospective single-center pilot study to investigate perioperative serum cortisol concentrations as our primary outcome measure in patients referred to curative surgery for central or adrenal CS. Patients did not receive any glucocorticoid replacement until the morning of the first postoperative day. In addition, we aimed to plot serum concentrations of cortisol and ACTH also during long-term outpatient visits to investigate the duration until the HPA axis recovers after surgery and glucocorticoid replacement can be discontinued. We recruited patients who underwent pituitary surgery for other reasons than CS as a control group.

## Methods

### Study design

This was a prospective pilot study conducted at the Medical University of Graz, Austria. The study’s main aim was to determine serum cortisol concentrations and clinical evidence of hypocortisolism in patients with CS who underwent curative surgery for central or adrenal CS without receiving glucocorticoid replacement until the first postoperative day. We furthermore planned to monitor patients’ serum cortisol concentrations as part of routine outpatient visits after surgery to investigate (a) if and when normal serum cortisol concentrations are accomplished after curative surgery, and (b) for how long postoperative glucocorticoid replacement is required. For comparison, we also observed serum cortisol concentrations in patients who underwent pituitary surgery for other reasons than CS.

The study was approved by the ethics committee of the Medical University of Graz, Austria (approval number: 29-083ex2016).

### Study participants

Eligible study participants for the CS group were patients with confirmed central or adrenal CS aged 18 years or older in whom curative surgery was planned as first-line treatment. We established the diagnosis of central or adrenal CS according to guidelines of the Endocrine Society [20] that require the confirmation of unsuppressed cortisol secretion by at least two of the following tests: urine free cortisol (at least two measurements), late-night salivary cortisol (two measurements), 1-mg overnight dexamethasone suppression test, or longer low-dose dexamethasone suppression test. We obtained a thorough patient history to rule out exogenous glucocorticoid exposure. To establish the cause of hypercortisolism, we performed serial measurements of ACTH with at least two measurements on separate days. Suppressed serum ACTH concentrations (<10 pg/mL) were considered indicative of ACTH-independent CS and prompted adrenal imaging (CT or MRI), inappropriately normal or elevated ACTH serum concentrations (>20 pg/mL) were considered suggestive of ACTH-dependent CS and were followed by pituitary imaging (MRI) [2]. In patients with inconclusive biochemical results or imaging studies (i.e., ACTH serum concentrations 10–20 pg/mL, no pituitary adenoma or pituitary adenoma <6 mm in MRI studies), we usually conduct additional biochemical tests (e.g., high dose dexamethasone suppression test, CRH test) and/or bilateral inferior petrosal sinus sampling (BIPSS) to rule out ectopic ACTH-dependent CS [2, 21]. All patients suffered from clinical CS and there was no patient considered to have mild autonomous cortisol secretion (MACS) according to our judgment. Patients were eligible to participate in the study group without CS if they were older than 18 years and scheduled for curative pituitary surgery for any other reason than CS. In this group, CS was ruled out clinically and/or biochemically before surgery [20].

Exclusion criteria for study participation were confirmed ectopic ACTH-dependent CS, intake of any medication interfering with glucocorticoid metabolism (e.g., ketoconazole, metyrapone, mitotane, fluconazole), or lack of informed consent.

Study participants were recruited from the Department of Endocrinology and Diabetology and from the Department of Neurosurgery at the Medical University of Graz, Austria. All study participants gave written informed consent prior to any study-related procedures.

### Primary outcome measure

The primary outcome measure was the proportion of patients with CS undergoing curative surgery without perioperative glucocorticoid replacement who had low postoperative serum cortisol concentrations (defined as serum cortisol below 5.0 µg/dL [138 nmol/L]).

### Secondary outcome measures

Secondary outcome measures included (a) differences between preoperative serum cortisol concentrations and postoperative measurements in patients with and without CS, (b) the postoperative interval until a serum cortisol concentration above 5.0 µg/dL (138 nmol/L) was reached in patients who were successfully treated for CS, and (c) the postoperative interval in which glucocorticoid replacement therapy was necessary in patients who were successfully treated for CS. We furthermore aimed to plot perioperative and postoperative serum cortisol concentration in patients with and without CS as well as serum ACTH concentrations in patients with CS. For safety reasons, patients were regularly monitored for possible clinical or biochemical signs of hypocortisolism (i.e., hypotension, hyponatremia, or clinical presentation of adrenal insufficiency according to the judgment of the treating physician).

### Procedures

Initial blood samplings (BS1) were performed preoperatively in the morning of the planned surgery between 6.30 and 8.00 a.m. after an overnight fast. We performed further blood samplings immediately postoperatively (BS2), in the evening of the day of surgery (BS3, between 7.00 and 8.00 p.m.), in the morning of the first postoperative day (BS4, between 7.00 and 8.00 a.m. after an overnight fast), and in the morning of the third postoperative day (BS5, between 7.00 and 8.00 a.m. after an overnight fast). Parameters of interest were either measured immediately after collection or immediately centrifuged and frozen until measurement the following day.

We measured the following parameters in every collected sample from BS1–5: cortisol, ACTH, thyroid-stimulating hormone (TSH), free thyroxin (fT4), free triiodothyronine (fT3), parathyroid hormone (PTH), 25-hydroxyvitamin D (25[OH]D), calcitriol, aldosterone, renin, sex hormone-binding globulin (SHBG), serum electrolytes (sodium, potassium, total and free calcium, magnesium), creatinine, plasma glucose, total cholesterol, high-density lipoprotein-cholesterol (HDL-cholesterol), low-density lipoprotein-cholesterol (LDL-cholesterol), and triglycerides. Furthermore, we obtained a venous blood gas test to detect changes in acid–base metabolism.

Immediately after BS4 (i.e., in the morning of the first postoperative day), patients received individualized oral glucocorticoid replacement with hydrocortisone, with dosage derived from body surface (calculated as 0.007184 × body height in cm^0.725^ × body weight in kg^0.425^). Patients received a total daily dose of 10–12 mg of hydrocortisone/m^2^ body surface divided into two daily administrations in the morning and the early afternoon, respectively (~2/3 of the total dose was administered in the morning, while ~1/3 of the daily dose was administered in the early afternoon). We performed BS5 before the planned intake of the morning dose of hydrocortisone.

**Fig. 1:**
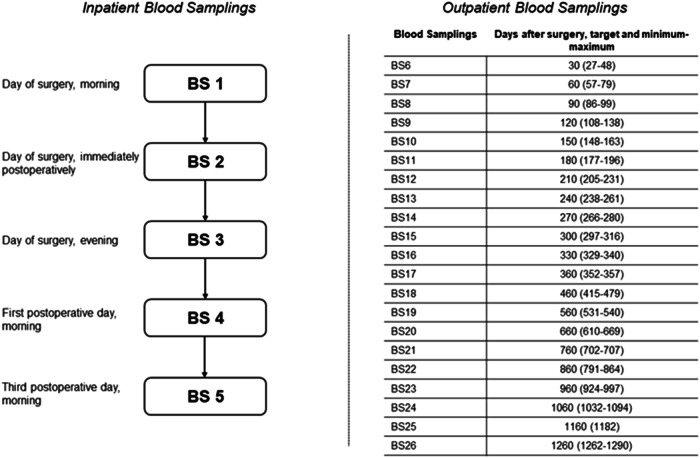
Flow-chart of inpatient blood samplings (BS1–5) and summary of timeframes of outpatient blood samplings (BS6–26). BS blood sampling

After discharge from inpatient treatment, patients were scheduled for routine visits at the outpatient clinic of the Department of Endocrinology and Diabetology at the Medical University of Graz. The timing of these outpatient visits was subject to the discretion of the treating physician and was dependent on clinical and biochemical findings during patient care. To utilize biochemical data gathered during these routine visits for study purposes, we categorized every blood sampling (BS) in dependence on the duration from the day of surgery. For the first year after surgery, we used intervals of 30 days to categorize data, i.e., BS6 took place ~30 days after surgery, BS7 ~60 days after surgery, and so on. After the first postoperative year, we used intervals of 100 days, since routine outpatient visits became less regular after this period. In cases where no biochemical data were available at a given time frame, data input was omitted and continued when next available (e.g., no routine outpatient visit 90 days after surgery, thus no data input for BS8). Even though the choice of biochemical tests during routine outpatient visits was made by the treating physician regardless of study participation, cortisol and ACTH were measured at each visit. Every measurement of cortisol and ACTH took place before the intake of any morning medication including glucocorticoids. Figure 1 shows a flow-chart of perioperative and postoperative blood samplings during inpatient treatment (BS1–5) as well as a summary of the timeframes of postoperative outpatient blood measurements (BS6–26).

### Measurements

Biochemical parameters of interest were either immediately measured after blood collection or, in case of samples that were collected in the evening, samples were immediately centrifuged and frozen, and biochemical parameters were measured the following day. We measured serum cortisol concentrations using a commercially available immunoassay (ADVIA Centaur; Siemens Healthcare Diagnostics Inc., Tarrytown, NY, USA), with intra-, inter-assay, and total coefficients of variation (CVs) ranging from 2.98–3.69, 3.99–5.45, and 4.98–6.58%, respectively. Serum cortisol concentrations were provided as µg/dL (multiply by 27.59 to convert µg/dL to nmol/L). ACTH was measured from EDTA-plasma by an Immulite ® 2000 ACTH chemiluminescent immunometric assay (Siemens Healthcare Diagnostics Inc., Tarrytown, NY, USA). The limit of detection for ACTH in plasma was 5 pg/mL, intra- and inter-assay CVs reached from 8.7 to 9.5% and 6.1 to 10.0%, respectively (for low to high plasma concentrations, i.e., 23–421 pg/mL).

### Data analysis

We analyzed data distribution using descriptive statistics and the Kolmogorov–Smirnov test. Continuous data with a normal distribution are shown as means with standard deviation (SD), while continuous data with a skewed distribution are shown as medians with interquartile range. Categorical data are presented as percentages. To evaluate our primary outcome measure, we used descriptive statistics to depict the proportion of postoperative low serum cortisol concentrations (defined as serum cortisol below 5.0 µg/dL [138 nmol/L]) and their time of occurrence. To compare baseline characteristics between patients with and without CS, we used Student’s *t*-test, Mann–Whitney *U* test, or Fisher’s exact test, depending on the scale of measure and data distribution. Wilcoxon signed-rank test was used to compare postoperative cortisol concentrations (BS2–5) with preoperative parameters (BS1). Differences in the time period until a serum cortisol concentration above 5.0 µg/dL (138 nmol/L) was reached and until glucocorticoid replacement therapy was discontinued were compared between patients with central and adrenal CS by Mann–Whitney *U* test. In graphical depictions of cortisol and ACTH measurements over time, missing data were interpolated to obtain continuous charts.

A two-sided *p* value < 0.05 was considered as statistically significant. We performed all statistical operations using IBM-SPSS software version 29 (IBM Corp., Armonk, NY, USA).

## Results

In total, 12 patients with CS as well as 6 patients who underwent pituitary surgery for other reasons were included in the study. Of the 12 patients with CS, 7 (58%) had central CS and 5 (42%) had adrenal CS. All patients with central CS underwent endoscopic transsphenoidal pituitary surgery, whereas four patients in the group with adrenal CS had unilateral adrenalectomy (using a laparoscopic approach in three patients and a transperitoneal approach in one patient) and one patient had adrenal radiofrequency ablation. All patients in the group with other forms of pituitary adenomas than central CS underwent endoscopic transsphenoidal pituitary surgery. The mean follow-up time in patients with CS was 876 ± 562 days and 859 ± 631 days in patients without CS.

**Fig. 2:**
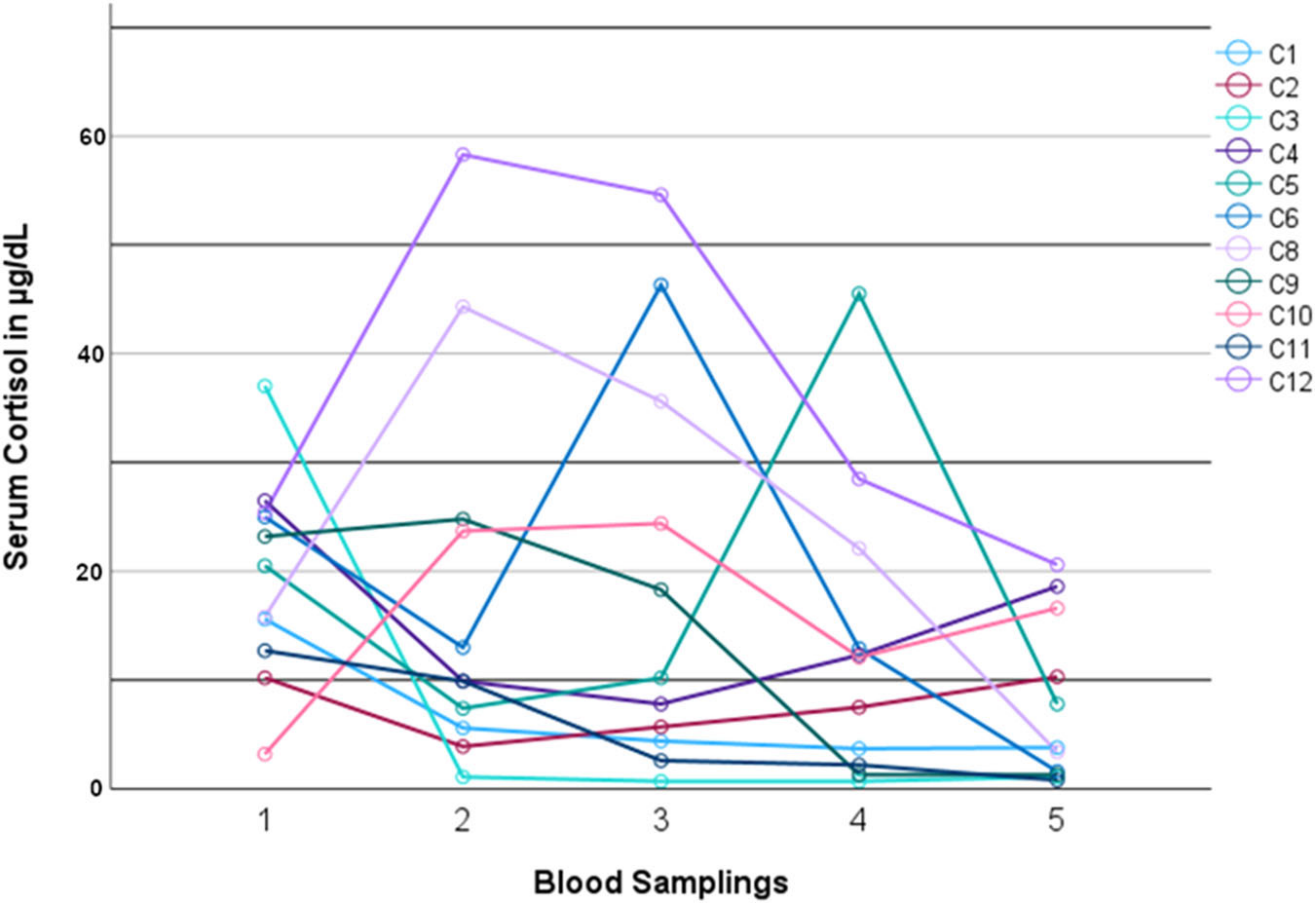
Serum cortisol concentration of patients with CS during inpatient treatment. C1–12 represents patients with CS. Blood sampling 1 took place on the morning of surgery, blood sampling 2 immediately after surgery, blood sampling 3 on the evening of the day of surgery, and blood sampling 4 and 5 on the morning of postoperative days 1 and 3, respectively. CS Cushing’s syndrome

Baseline characteristics of all study participants in the morning of the planned intervention (BS1) are shown in Table 1. Figure 2 shows the serum cortisol concentration in patients with CS across the blood samplings during inpatient treatment (BS1–5). To improve legibility, data from one patient (C7) is not included in the figure due to an excessively high postoperative serum cortisol (peak serum cortisol at BS3: 151.9 µg/dL [4191 nmol/L]). Patients with CS had a significantly lower serum cortisol concentration on the third postoperative day (BS5, serum cortisol: 8.5 ± 7.6 µg/dL [235 ± 210 nmol/L]) when compared to their initial cortisol levels on the day of intervention (BS1, mean serum cortisol: 19.9 ± 8.9 µg/dL [549 ± 246 nmol/L], *p* = 0.023). Furthermore, patients without CS showed significantly higher serum cortisol concentrations immediately postoperatively, in the evening of the day of surgery, as well as on the third postoperative day when compared to preoperative values (baseline BS1 mean serum cortisol: 15.9 ± 4.8 µg/dL [439 ± 132 nmol/L], BS2 mean serum cortisol: 31.2 ± 7.2 µg/dL [861 ± 199 nmol/L], BS3 mean serum cortisol: 34.3 ± 9.1 µg/dL [946 ± 251 nmol/L], and BS5 mean serum cortisol: 18.7 ± 3.0 µg/dL [516 ± 83 nmol/L], *p* = 0.028, *p* = 0.046, and *p* = 0.046, respectively). All other measured serum cortisol concentrations during inpatient treatment did not differ significantly compared to baseline in both patient groups.

**Table 1:** Baseline characteristics of patients with and without CS

Characteristics	All patients (*n* = 18)	CS (*n* = 12)	No CS (*n* = 6)	*p* value
Age (years)	47.0 ± 15.2	43.5 ± 16.9	54.0 ± 8.3	0.096
Female gender (%)	66.7	75	50	0.344
Cortisol (µg/dL)	18.6 ± 7.9	19.9 ± 8.9	15.9 ± 4.8	0.331
Cortisol (nmol/L)	513 ± 218	549 ± 246	439 ± 132	
ACTH (pg/mL)	30.0 (5.0–54.6)		28.6 (22.0–80.5)	
Central CS		46.7 (40.8–66.6)		0.628
Adrenal CS		5.0 (5.0–5.0)^a^		0.004
TSH (µU/mL)	1.46 ± 0.86	1.34 ± 0.84	1.70 ± 0.93	0.420
fT4 (pmol/L)	14.3 ± 3.4	14.5 ± 3.1	13.8 ± 4.2	0.703
fT3 (pmol/L)	4.0 ± 0.8	3.8 ± 0.7	4.5 ± 1.0	0.140
PTH (pg/mL)	43.7 (32.6–53.3)	41.7 (32.5–51.6)	46.3 (33.5–67.1)	0.494
25(OH)D (ng/mL)	24.1 (12.6–29.3)	21.9 (12.2–31.8)	25.7 (14.0–29.2)	0.892
Calcitriol (pmol/L)	120.5 (77.3–144.3)	102.5 (68.3–131.8)	135.5 (114.5–176.8)	0.102
Aldosterone (ng/dL)	5.0 (3.7–8.6)	3.8 (3.7–7.2)	9.2 (4.7–15.7)	0.037
Renin (µU/mL)	11.9 (2.3–29.1)	13.4 (3.4–47.9)	11.9 (1.8–29.1)	0.750
SHBG (nmol/L)	31.7 (16.7–47.3)	27.3 (16.4–40.5)	35.6 (17.6–59.1)	0.437
Serum sodium (mmol/L)	141 ± 2	141 ± 3	140 ± 2	0.200
Serum potassium (mmol/L)	4.0 (3.8–4.3)	3.9 (3.6–4.4)	4.0 (3.8–4.1)	0.750
Serum total calcium (mmol/L)	2.34 (2.29–2.40)	2.33 (2.27–2.38)	2.37 (2.31–2.51)	0.180
Serum free calcium (mmol/L)	1.19 (1.16–1.21)	1.18 (1.13–1.20)	1.20 (1.16–1.26)	0.250
Serum magnesium (mmol/L)	0.89 ± 0.09	0.90 ± 0.10	0.87 ± 0.06	0.399
Creatinine (mg/dL)	0.91 ± 0.23	0.85 ± 0.24	1.05 ± 0.12	0.107
Plasma glucose (mg/dL)	92 ± 15	88 ± 16	100 ± 6	0.121
Total cholesterol (mg/dL)	204 ± 59	188 ± 54	236 ± 60	0.110
HDL-cholesterol (mg/dL)	46 (39–54)	46 (40–54)	43 (34–72)	0.583
LDL-cholesterol (mg/dL)	130 (73–154)	113 (71–140)	154 (112–189)	0.180
Triglycerides (mg/dL)	153 (109–201)	143 (106–189)	198 (111–250)	0.213
Blood pH	7.39 ± 0.05	7.39 ± 0.06	7.39 ± 0.02	0.776

Data are shown as means with standard deviations, medians with interquartile range or percentages, as appropriate. Comparisons between the patient groups with and without CS were performed using unpaired Student’s *t*-test, Mann–Whitney *U* test, or Fisher’s exact test, as appropriate

*25(OH)D* 25-hydroxyvitamin D, *ACTH* adrenocorticotrophic hormone, *CS* Cushing’s syndrome, *fT3* free triiodothyronine, *fT4* free thyroxine, *HDL-cholesterol* high-density lipoprotein-cholesterol, *LDL-cholesterol* low-density lipoprotein-cholesterol, *PTH* parathyroid hormone, *SHBG* sex hormone-binding globulin, *TSH* thyroid-stimulating hormone

^a^5 pg/mL represents the lower detection limit of the ACTH assay, all patients with adrenal CS had baseline ACTH serum concentrations <5 pg/mL

Table 2 shows the number and proportion of patients who developed serum cortisol concentrations below 5.0 µg/dL (138 nmol/L) during inpatient treatment (BS1–5). Of note, no patient without CS showed a serum cortisol below 5.0 µg/dL (138 nmol/L) at any time, while the proportion of patients with low cortisol concentrations postoperatively gradually increased over time in patients with CS (Table 2). No patient developed clinical or biochemical signs of hypocortisolism during inpatient treatment.

**Table 2:** Number and proportion (%) of patients who showed serum cortisol concentrations below 5.0 µg/dL (138 nmol/L) during inpatient care (BS1–5)

	BS1	BS2	BS3	BS4	BS5
All patients (*n* = 18)	1 (6%)	2 (11%)	3 (17%)	4 (22%)	6 (33%)
Central CS (*n* = 7)	1 (14%)	0 (0%)	1 (14%)	2 (29%)	4 (57%)
Adrenal CS (*n* = 5)	0 (0%)	2 (40%)	2 (40%)	2 (40%)	2 (40%)
No CS (*n* = 6)	0 (0%)	0 (0%)	0 (0%)	0 (0%)	0 (0%)

*BS* blood sampling, *CS* Cushing’s syndrome

**Fig. 3:**
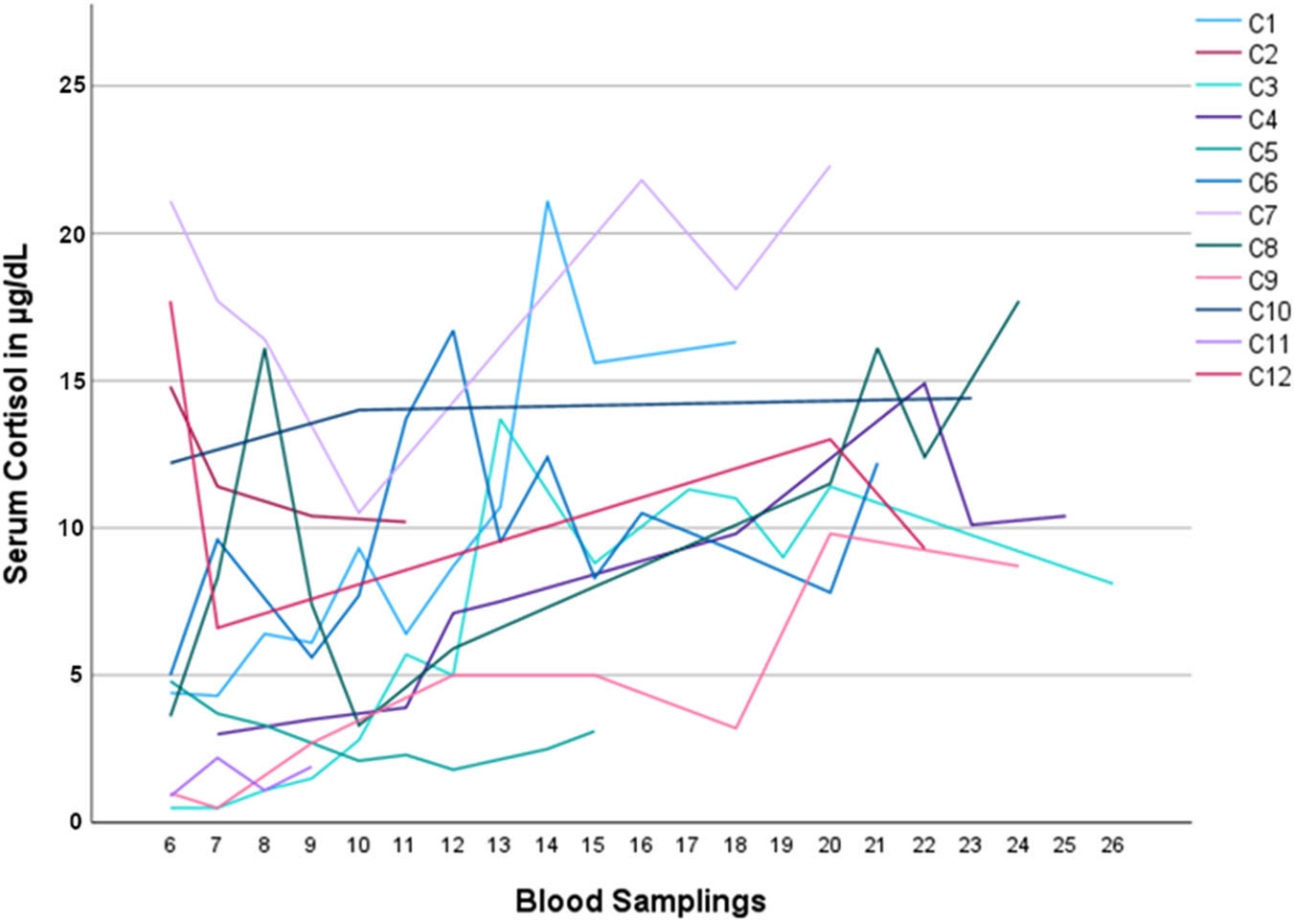
Serum cortisol concentrations of patients with CS during follow-up visits after discharge from inpatient treatment. C1–12 represents patients with CS. Blood samplings 6–26 took place during routine outpatient visits after surgery, please refer to Fig. 1 for target time frame of blood samplings. CS Cushing’s syndrome

**Fig. 4:**
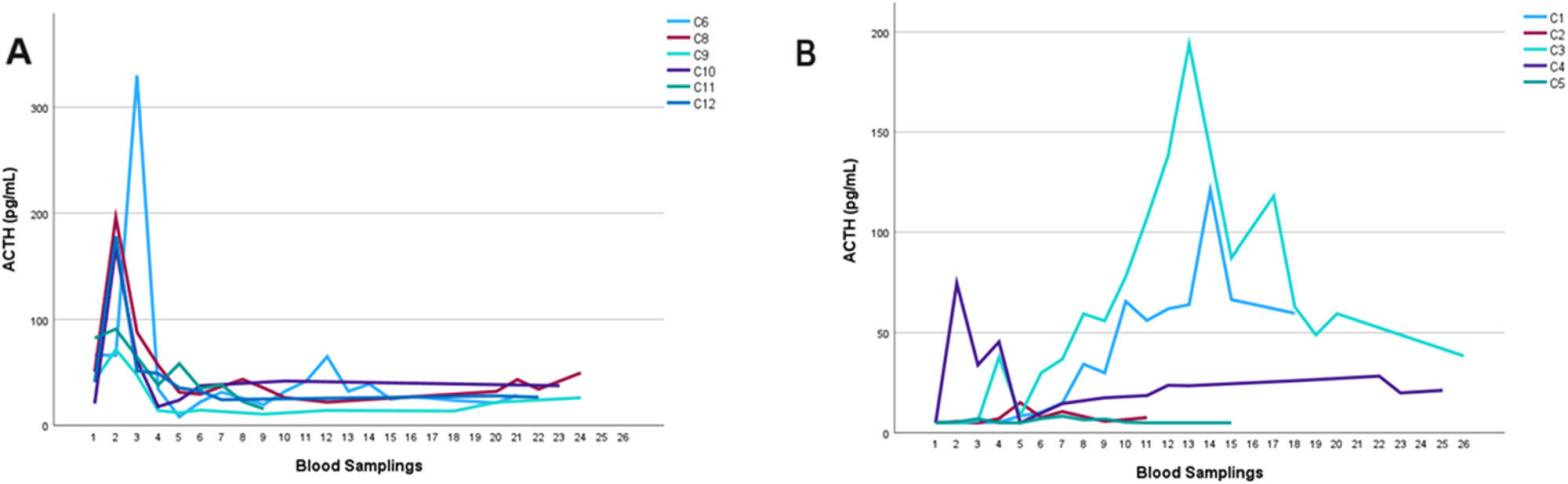
Serum ACTH concentrations in patients with central (**A**) and adrenal (**B**) CS during inpatient and outpatient treatment. C1–12 represents patients with CS. Blood sampling 1 took place on the morning of surgery, blood sampling 2 immediately after surgery, blood sampling 3 on the evening of the day of surgery, and blood sampling 4 and 5 on the morning of postoperative days 1 and 3, respectively. Blood samplings 6–26 took place during routine outpatient visits after surgery, please refer to Fig. 1 for target time frame of blood samplings. ACTH adrenocorticotrophic hormone, CS Cushing’s syndrome

Patients with CS were discharged from inpatient treatment after 11 ± 7 days with a mean hydrocortisone replacement dose of 26 ± 13 mg/day. No patient without CS required postoperative glucocorticoid replacement. Furthermore, two patients with central CS were discharged without glucocorticoid therapy due to biochemical and/or clinical evidence for persistent postoperative hypercortisolism during their hospital stay. Figure 3 shows all available serum cortisol parameters in participants with CS measured during any follow-up visits after discharge. Figure 4 portrays ACTH serum concentrations during inpatient and outpatient treatment in patients with central and adrenal CS. In patients with CS who received hydrocortisone replacement after inpatient treatment (*n* = 10), the mean time until the first occurrence of serum cortisol concentration above 5.0 µg/dL (138 nmol/L) was 129 ± 97 days and hydrocortisone replacement could be discontinued after a mean time of 402 ± 243 days. Times until first serum cortisol above 5.0 µg/dL (138 nmol/L) and until discontinuation of hydrocortisone were not significantly different between patients with central and adrenal CS (*p* = 0.564 and *p* = 0.773, respectively). There were no patients with CS who required permanent glucocorticoid replacement, however, two patients were lost to follow-up when they were still taking hydrocortisone and before a fasting serum glucocorticoid concentration above 5.0 µg/dL (138 nmol/L) was recorded (patients C5 and C11, lost to follow-up after 314 days and 123 days, respectively).

Serum cortisol concentrations of patients without CS during both inpatient treatment and follow-up visits are shown in Supplementary Fig. S1. Supplementary Fig. S2 portrays the serum cortisol concentrations after discharge from inpatient treatment in patients with CS who had no biochemical and/or clinical evidence for persistent postoperative hypercortisolism (*n* = 10).

No patient developed venous thromboembolism due to hypercortisolism throughout the entire observation period.

## Discussion

In patients with CS receiving no glucocorticoid replacement after surgery until the first postoperative day, we found a low proportion of patients with CS (11%) who developed serum cortisol concentrations below 5.0 µg/dL (138 nmol/L) immediately after surgery (BS2), while this proportion increased continuously over the subsequent postoperative days. No patient developed any clinical or biochemical signs of hypocortisolism during inpatient treatment.

Our findings suggest that perioperative glucocorticoid replacement in curative surgery for CS may be safely omitted until the first postoperative day, a notion that is supported by previous investigations [18, 19]. In a review on the perioperative management of patients with CD, Varlamov et al. [14] suggested to withhold perioperative glucocorticoids unless patients had been pre-treated with medical therapy, were clinically unstable, or had any surgical complication. They furthermore recommended to delay glucocorticoids for up to 72 h after surgery to allow remission assessment [14]. Withholding perioperative glucocorticoids until the first postoperative day (or even longer) may simplify clinical care of patients and may also avoid potential harms of glucocorticoid treatment during this time. We are aware that the risk of short-term glucocorticoid treatment is likewise very low but potential adverse effects cannot be excluded entirely [22, 23].

In our study, we observed, as expected, a significant decline in serum cortisol concentrations in patients with CS after surgery. In general, postoperative hypocortisolism is considered a positive predictor for surgical remission: in a study by Costenaro et al. [19], a serum cortisol nadir ≤5.7 µg/dL (157 nmol/L) within 10–12 days after pituitary surgery had a positive predictive value of 100% and a negative predictive value of 78% for CD remission. A retrospective study in 257 patients with CD reported that achieving serum cortisol concentrations <2 µg/dL (55 nmol/L) within 21 h after surgery appeared to accurately predict durable remission in the intermediate term [24]. It must be noted that there is no universal definition of surgical remission in CS, however, serum cortisol concentrations below 5.0 µg/dL (138 nmol/L) during the first 3 postoperative days are usually considered as early remission while sustained remission is defined as need for glucocorticoid replacement for at least 6 months after surgery [25, 26].

Due to the postoperative suppression of the HPA axis after glucocorticoid excess due to CS is resolved, all patients require mandatory glucocorticoid replacement [9]. Patients must be educated about the possible occurrence of glucocorticoid withdrawal syndrome, that may develop 3–10 days postoperatively despite appropriate glucocorticoid replacement as well as about measures for the prevention and treatment of adrenal crisis [27–29]. In contrast to patients with CS, serum cortisol concentrations in our control group without CS even significantly increased following surgery in a physiological manner. No patient developed hypocortisolism during inpatient treatment or the following outpatient care.

Long-term cortisol concentrations after discharge from inpatient treatment portrayed a broad heterogeneity among patients with CS. We documented first signs of HPA axis recovery after 129 ± 97 days (defined as the first occurrence of a serum cortisol concentration above 5.0 µg/dL [138 nmol/L] prior to the morning intake of glucocorticoid replacement), while glucocorticoid replacement was discontinued after 402 ± 243 days, indicating a complete functional HPA recovery. This is in line with previous findings on the postoperative period in patients with CS, where HPA axis recovery took 15–17 months after surgery for CD and 19–30 months after surgical therapy for adrenal CS [27, 30–32]. However, possibly due to the low sample sizes, we were unable to find a significant difference in recovery durations between central and adrenal CS in our cohort. No patient required permanent glucocorticoid replacement, however, two patients were lost to follow-up while they were still on hydrocortisone replacement. Interestingly, we found an exaggerated postoperative increase in ACTH serum concentrations in some patients who were treated for adrenal CS. For comparison, ACTH levels declined after the inpatient treatment period in patients with central CS and stayed mostly within the normal range during outpatient follow-up.

We found no significant differences in preoperative serum cortisol concentrations between patients with and without CS. However, basal serum cortisol levels are generally considered inappropriate to establish the diagnosis of CS, since there is a relevant overlap between individuals with and without endogenous glucocorticoid excess and CS is mostly characterized by the abolition of the cortisol circadian rhythm and not absolute morning cortisol levels per se [20, 33]. As preoperative blood sampling took place on the morning of surgery, one could also hypothesize that a cortisol stress response induced by surgery influenced our findings [34]. However, we must also acknowledge that our study population size may be too small to draw statistically firm conclusions when comparing patients with and without CS and these comparisons are, therefore, probably prone to statistical type 2 errors. Low study population size may also explain why we could not detect characteristic metabolic complications in CS (e.g., hyperlipidemia).

Our study has some limitations that should be noted when interpreting its findings. While data are available from all participants during the inpatient period (BS1–5), subsequent outpatient visits were scheduled independently from study participation at the discretion of the treating physician. Consequently, data are not available in each patient at all given time points during outpatient care, thus limiting our findings regarding the outpatient period. We unfortunately did not measure dehydroepiandrosterone-sulfate (DHEA-S), a parameter that may be useful in the diagnosis of hypercortisolism and the differentiation between central and adrenal CS [35, 36]. As already mentioned, our study’s sample size may be too small to find statistically significant differences between subgroups. Thus, baseline differences between patients with and without CS should not be overstated in particular (e.g., differences in plasma aldosterone). Another limitation is the use of routine immunoassays to measure steroid hormones (including serum cortisol) instead of standardized mass spectrometry steroid assays [37]. We have to acknowledge that we did not include patients with ectopic CS so that our findings cannot be uncritically applied to this patient group. In our clinical routine, we apply the same procedures regarding perioperative glucocorticoid therapy in patients with ectopic CS as in this study, but given the usually severe hypercortisolism in these patients, they may require higher postoperative glucocorticoid doses depending on clinical judgment. Last, as a single-center study, our results may not be generalizable to other tertiary care centers. The strengths of our study include its well-defined study population of patients with CS and the long mean follow-up duration of more than 2 years. Thus, our study provides unique insights into the changes of the HPA axis after the resolution of glucocorticoid excess. To the best of our knowledge, no previous postoperative study has specifically addressed the course of serum cortisol concentrations at multiple time points during inpatient care as a primary outcome measure in patients undergoing surgery for hypercortisolism while receiving no glucocorticoid replacement until the morning of the first postoperative day. Finally, our study’s results may be useful in creating and implementing perioperative standards of care in CS.

In conclusion, in our study of patients undergoing surgery for CS, there were no safety concerns for withholding glucocorticoid replacement until the morning of the first postoperative day, suggesting that this approach represents a reasonable clinical strategy. Cortisol serum concentrations significantly decreased after surgery in patients with CS, while we saw an increase in cortisol levels in patients without CS. Patients with CS required postoperative glucocorticoid replacement for a mean period of 402 ± 243 days, and no patient with CS required permanent glucocorticoid therapy. We did not observe any sign of secondary adrenal insufficiency in patient without CS during the entire study duration. The results of this pilot study should be confirmed in future larger prospective trials.

### Supplementary information

Supplementary Information

## Data Availability

The data that support the findings of this study are not openly available due to reasons of sensitivity and are available from the corresponding author upon reasonable request.
