# Supplementary material for: No requirement of perioperative glucocorticoid replacement in patients with endogenous Cushing’s syndrome – a pilot study
Source: Endocrine. 2024 Apr 22;85(2):926–36. doi: 10.1007/s12020-024-03832-1 (PMC11291516; doi:10.1007/s12020-024-03832-1)
Supplement: Supplementary file 1 — Supplementary Information [file 12020_2024_3832_MOESM1_ESM.docx]

**Supplementary Information**

*Endocrine*

**No requirement of perioperative glucocorticoid replacement in patients with endogenous Crushing’s syndrome - a pilot study**

*Christian Trummer^1^, Marlene Pandis^1^, Verena Theiler-Schwetz^1^, Lisa Schmitt^1^, Barbara Obermayer-Pietsch^1,2^, Verena Gellner^3^, Andrea Simon^4^, Stefan Pilz^1^*

^1^Division of Endocrinology and Diabetology, Department of Internal Medicine, Medical University of Graz, Graz, Austria; ^2^Endocrinology Lab Platform, Department of Internal Medicine and Department of Gynecology and Obstetrics, Medical University of Graz, Graz, Austria; ^3^Department of Neurosurgery, Medical University of Graz, Graz; ^4^ Division of General, Visceral, and Transplantation Surgery, Department of Surgery, Medical University of Graz, Graz, Austria

**Correspondence:**

Christian Trummer, MD, PhD

Division of Endocrinology and Diabetology

Department of Internal Medicine

Medical University of Graz

Auenbruggerplatz 15, 8036 Graz, Austria

Tel: +43 316 385 12383

Fax: +43 316 385 13428

Email: christian.trummer@medunigraz.at

**Supplemental Fig. 1** Serum cortisol concentrations in patients without CS during inpatient and outpatient measurements


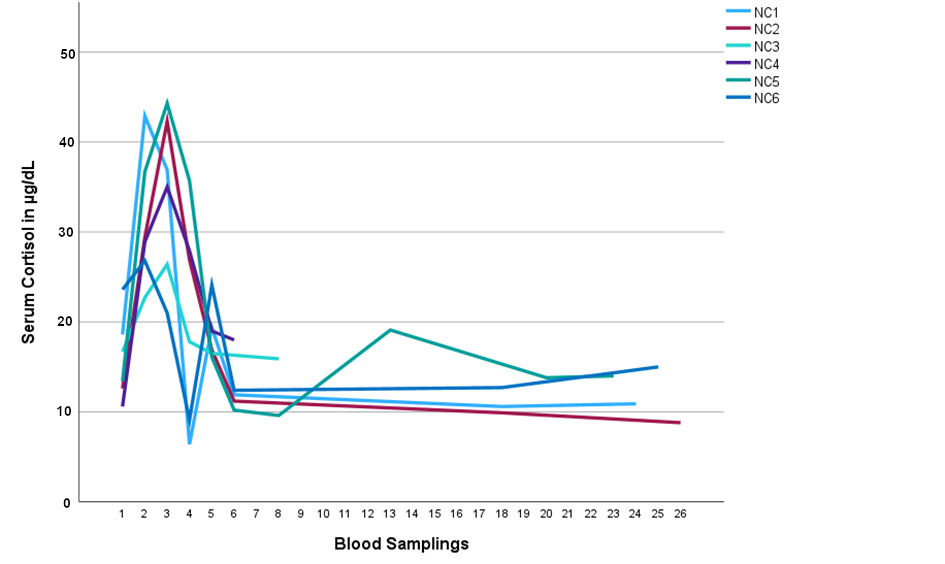


NC1-6 represents patients without Cushing’s syndrome. Blood sampling 1 took place on the morning of surgery, blood sampling 2 immediately after surgery, blood sampling 3 on the evening of the day of surgery, blood sampling 4 and 5 on the morning of postoperative day one and three, respectively. Blood samplings 6-26 took place during routine outpatient visits after surgery, please refer to Fig. 1 for target time frame of blood samplings.

**Supplemental Fig. 2** Serum cortisol concentrations after discharge from inpatient treatment in patients who were successfully treated for CS (n=10)


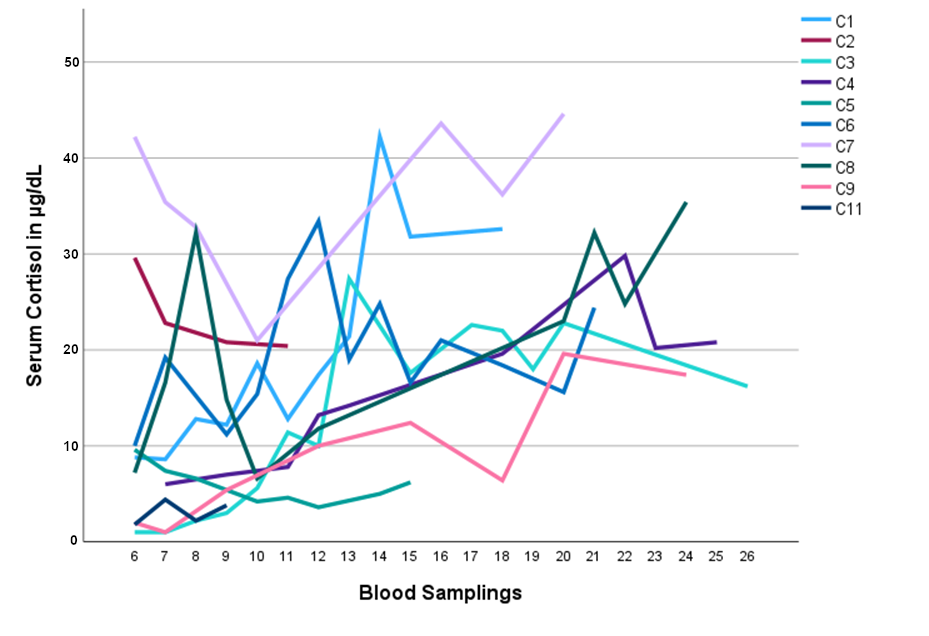


C1-11 represents patients with Cushing’s syndrome. Blood samplings 6-26 took place during routine outpatient visits after surgery, please refer to Figure 1 for target time frame of blood samplings. CS = Cushing’s syndrome
